# Levodopa‐Responsive Parkinsonism Preceded by Isolated Upper Limb Tremor Associated with *PTEN* Variant

**DOI:** 10.1002/mdc3.70844

**Published:** 2026-09-28

**Authors:** Sacha E. Gandhi, Rekha Siripurapu, Audrey Smith, Christopher Kobylecki

**Affiliations:** ^1^ Department of Neurology Institute of Neurological Sciences Glasgow UK; ^2^ Department of Neuroradiology, Manchester Centre for Clinical Neurosciences Northern Care Alliance NHS Foundation Trust Salford UK; ^3^ Manchester Centre for Genomic Medicine Manchester University NHS Foundation Trust Manchester UK; ^4^ Department of Neurology, Manchester Centre for Clinical Neurosciences Northern Care Alliance NHS Foundation Trust Salford UK; ^5^ Division of Neuroscience, Faculty of Biology, Medicine and Health University of Manchester Manchester UK

**Keywords:** genetic, leukodystrophies, parkinsonism

PTEN hamartoma tumor syndromes (PHTS) due to variants in the *PTEN* gene lead to disordered cellular proliferation, developmental abnormalities and malignancy. The expanding spectrum of neurological disorders associated with PHTS is increasingly recognized. We previously reported a case of PHTS with isolated upper limb action tremor, leukoencephalopathy and basal ganglia calcification.^1^ Follow up has now revealed hitherto unreported levodopa‐responsive parkinsonism. We discuss here the potential role of *PTEN* in the pathogenesis of parkinsonism, and expand the spectrum of reported neurological features of PHTS.

## Case Report

We report a man presenting with a left upper limb action tremor and mild dystonic posturing starting at the age of 33. This had been slowly progressive with no evidence of parkinsonism. He and his daughter both showed macrocephaly; his daughter had a history of developmental delay and a laryngeal haemagioma, with leukoencephalopathic changes on MR brain.^1^

He reported a gradual worsening of predominantly left upper limb action tremor, causing difficulty with drinking, using eating utensils and holding his mobile phone. When reviewed at the age of 46, he had a moderate left upper limb rest tremor persisting with posture holding, with a mild right upper limb postural tremor. There was no limb rigidity, ataxia or bradykinesia although tremor interfered with left‐sided finger tapping. His gait was normal.

His tremor worsened over the next two years, without definitive signs of parkinsonism. He did not tolerate treatment with propranolol or topiramate. He had several focal seizures with secondary generalization, treated only with lamotrigine.

When reviewed at age 49, he reported slowness of movement and occasional vivid dreams with enactment, although he did not undergo formal polysomnography. There were no bladder or bowel symptoms, orthostatic light‐headedness or falls. On examination, he had moderate facial hypomimia and normal eye movements with no axial rigidity. He had a moderate left greater than right upper limb rest tremor, persistent during posture holding. He had mild bradykinesia on the right upper limb and marked bradykinesia on the left, without rigidity (Video 1). He walked with an absent arm swing and some start hesitation; he took three steps back on the pull test but was able to right himself.

**Video 1:** At diagnosis of parkinsonism prior to levodopa treatment. Video shows facial hypomimia, rest and action tremor bilaterally worse on the left, mild bradykinesia on the right and moderate‐marked bradykinesia on the left upper limb. Examination of gait shows reduced stride length and arm swing with some hesitation on turning.

He commenced levodopa/benserazide and gradually titrated the dose to 100/25 mg qds, with significant improvements in tremor, limb bradykinesia and gait (Video 2), and preserved postural reflexes.

**Video 2:** Established on levodopa/benserazide 100/25 mg qds. Video shows improvement in tremor, and significant improvement in bradykinesia which is absent on right side and only mild on left side. Gait assessment shows improved stride length and speed, and turns are more fluent.

MR brain showed progression of the leukoencephalopathic changes (Fig. 1). [123‐I]‐Ioflupane SPECT showed bilaterally reduced striatal uptake, the right side being more affected (Fig. 2). WGS panel did not identify any other genes known to be associated with parkinsonism and confirmed a likely pathogenic variant c.809 T>C p.(Met270Thr) in the NM_000314.8(*PTEN*) gene (Supporting Information).

**Figure 1:**
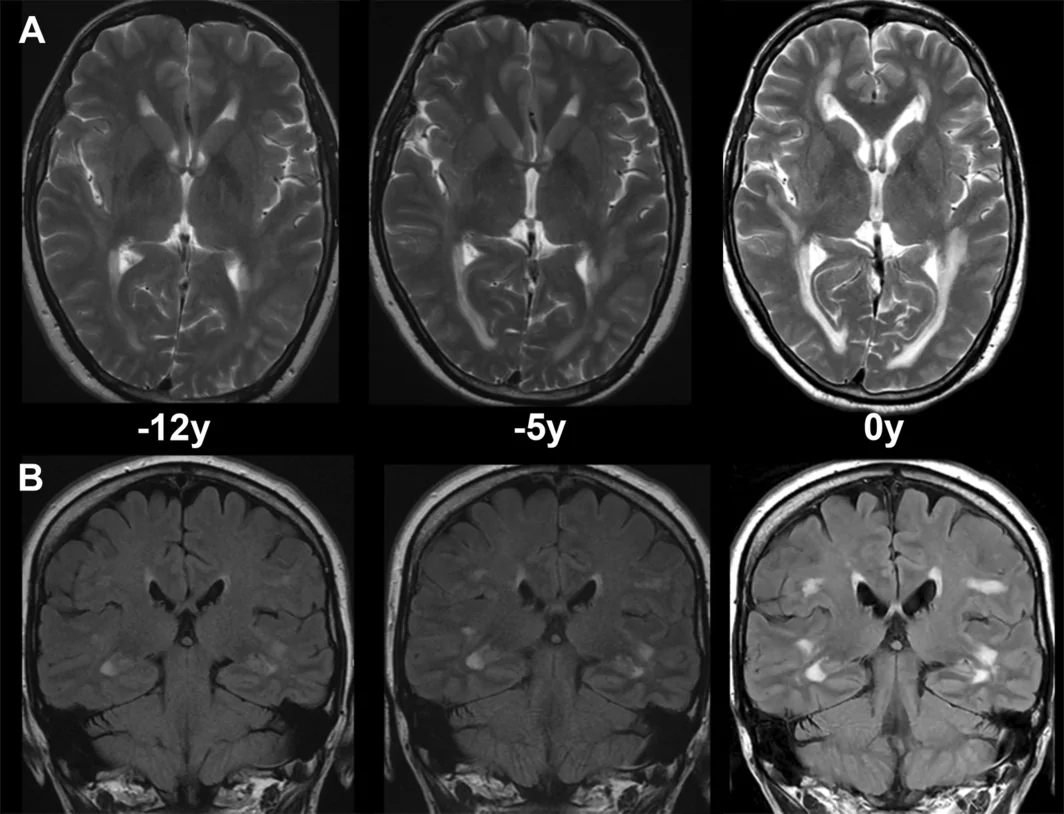
(A) Axial T2 and (B) Coronal Flair MR images show marked progression of white matter high signal intensity changes compared with 12 years and five years prior to presentation with parkinsonism, with interval white matter volume loss. There are no comparable basal ganglia signal changes or volume loss.

**Figure 2:**
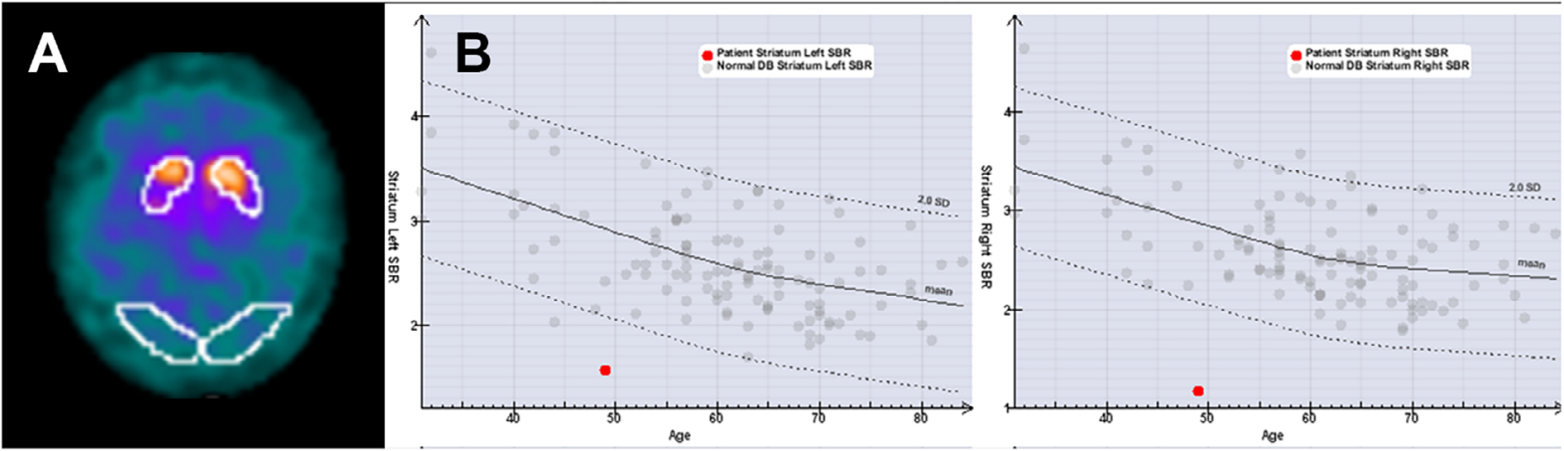
(A) Axial [^123^]‐ioflupane SPECT image showing reduced tracer activity in the striata bilaterally, more so on the right. (B) The DaTQUANT analysis confirmed significantly reduced uptake in the striata bilaterally.

## Discussion

PHTS are autosomal dominant conditions, due to pathogenic germline variants in the *PTEN* gene. As a tumor suppressor, PTEN regulates cellular growth and division.^2^ Consequently, increased cancer susceptibility occurs in PHTS, with increased risk and earlier age at onset for breast, thyroid, skin, colon, kidney and endometrial tumors.^3^

However, the clinical phenotype of PHTS continues to expand^4^ beyond oncological risks. Macrocephaly and autism spectrum disorder (ASD) are strongly associated with PHTS, while other neurological features include epilepsy, movement disorders and cortical abnormalities–such as focal cortical dysplasia, pachygyria, polymicrogyria and periventricular gliosis.^4^, ^5^ Our patient had focal epilepsy although had no obvious causative dysplastic abnormalities.

Movement disorders remain rare. Postural and action upper limb tremor was reported in a 57‐year‐old woman with PHTS, in association with deep gray calcification on MRI brain and prefrontal cortex, brainstem and cerebellar hypometabolism on FDG‐PET.^6^ We propose that parkinsonism represents an additional novel feature of the expanding PHTS phenotype, evidenced by the development of levodopa‐responsive parkinsonism with abnormal dopaminergic imaging in our patient with a pathogenic *PTEN* mutation. His initial tremor was interpreted as a dystonic‐type tremor but given subsequent progression we propose that it was likely to have represented an early atypical tremulous presentation of parkinsonism. We did not identify any features of cortical myoclonus although formal neurophysiology studies were not done. The possibility of co‐existent idiopathic PD cannot be excluded, while the good response to levodopa and absence of supporting features would make an atypical parkinsonian disorder less likely.

A biological link between parkinsonism and PHTS is strongly supported by the dual role of PTEN in regulating the PI3K/AKT/mTOR (PAM) pathway and the expression of PTEN‐induced kinase 1 (*PINK1*). Mechanistically, PTEN dephosphorylates PIP_3_ to PIP_2_, preventing AKT activation and suppressing the PAM pathway's protective effect against iron‐dependent cell death (ferroptosis), thereby augmenting PD risk.^7^ Furthermore, while *PINK1* mutations cause familial young‐onset autosomal recessive PD by disrupting mitochondrial signaling, *PTEN* loss‐of‐function mutations achieve a similar biological endpoint by downregulating *PINK1* expression. This downregulation leads to mitochondrial dysfunction and impaired clearance of damaged mitochondria.^8^ Basal ganglia calcification also represents a potential mechanism for the development of parkinsonism.

In conclusion, our findings expand the clinical associations of PHTS to include parkinsonism. Further reports of movement disorders in PHTS would help to strengthen this association, which has plausible biological mechanisms. The diagnosis of PHTS in neurological conditions also raises the important possibility of screening for treatable malignancies, which has been offered to those reported in this case.

## Author Roles

(1) Research project: A. Conception, B. Organization, C. Execution; (2) Manuscript Preparation: A. Writing of the first draft, B. Review and Critique

S.E.G.: 1B, 1C, 2A, 2B

R.S.: 1C, 2B

A.S.: 1C, 2B

C.K.: 1A, 1A, 1C, 2A, 2B

## Disclosures

**Ethical Compliance Statement:** Written informed consent for publication including video footage was obtained from the patient. As this was not a formal research study IRB approval was not required. We confirm that we have read the Journal's position on issues involved in ethical publication and affirm that this work is consistent with those guidelines.

**Financial Disclosures and Conflicts of Interest:** We have no conflicts of interest relating to this work. Author disclosures are available in the Supporting Information.

## Supporting information


**Data S1.** COI_disclosure.

**Data S2.** Supporting Information.

## Data Availability

Data sharing not applicable to this article as no datasets were generated or analysed during the current study.

## References

[1] Gandhi SE , Murphy HR , Kellett MW , Siripurapu R , Kobylecki C . Novel PTEN mutation with leukoencephalopathy, basal ganglia calcification and action tremor. Parkinsonism Relat Disord 2016;28:163–165. 10.1016/j.parkreldis.2016.04.003 27066848

[2] Nelen MR , van Staveren WC , Peeters EA , et al. Germline mutations in the PTEN/MMAC1 gene in patients with Cowden disease. Hum Mol Genet 1997;6(8):1383–1387. 10.1093/hmg/6.8.1383 9259288

[3] Yehia L , Plitt G , Tushar AM , et al. Longitudinal analysis of cancer risk in children and adults with germline PTEN variants. JAMA Netw Open 2023;6(4):e239705. 10.1001/jamanetworkopen.2023.9705 PMC10126871 37093598

[4] Dhawan A , Baitamouni S , Liu D , Eng C . Clinical neurologic features and evaluation of PTEN hamartoma tumor syndrome: a systematic review. Neurology 2024;103(7):e209844. 10.1212/WNL.0000000000209844 39250745

[5] Dhawan A , Baitamouni S , Liu D , et al. Exploring the neurological features of individuals with germline PTEN variants: a multicenter study. Ann Clin Transl Neurol 2024;11(5):1301–1309. 10.1002/acn3.52046 PMC11093251 38501559

[6] Grangeret J , Frismand S , Muller M , Renaud M , Verger A . Brain 18F‐FDG PET in Cowden syndrome. Clin Nucl Med 2022;47(2):e118–e119. 10.1097/RLU.0000000000004025 35006113

[7] Yang X , Liu T , Cheng H . PTEN: a new dawn in Parkinson's disease treatment. Front Cell Neurosci 2025;19:1497555. 10.3389/fncel.2025.1497555 PMC11931041 40129459

[8] Agarwal S , Muqit MMK . PTEN‐induced kinase 1 (PINK1) and parkin: unlocking a mitochondrial quality control pathway linked to Parkinson's disease. Curr Opin Neurobiol 2022;72:111–119. 10.1016/j.conb.2021.09.005 34717133

