# Supplementary material for: Levodopa‐Responsive Parkinsonism Preceded by Isolated Upper Limb Tremor Associated with PTEN Variant
Source: Mov Disord Clin Pract. 2026 Sep 28:10.1002/mdc3.70844. Online ahead of print. doi: 10.1002/mdc3.70844 (PMC13620421; doi:10.1002/mdc3.70844)
Supplement: Supplementary file 2 [file MDC3-9999-0-s002.docx]

**Supplementary material**

HGVS description: NM_000314.8(PTEN):c.809T>C p.(Met270Thr)

Classification: Likely pathogenic

Genomic coordinates: Chr10(GRCh38):g.87960901T>C

Evidence for variant classification using ACGS best practice guidelines 2024 (<https://www.acgs.uk.com/quality/best-practice-guidelines/>).

Key: Evidence code_Level

PM2_Moderate

Absent in control individuals from the gnomAD dataset (The Genome Aggregation Database, gnomAD v2.1.1 and v4.0, Cambridge, MA.

PP2_Supporting

PTEN has a low rate of benign missense variation, and missense variants in PTEN are a common mechanism of disease (Human Gene Mutation Database (HGMD) from BIOBASE Corporation ([www.biobase-international.com/hgmd](http://www.biobase-international.com/hgmd)).

PS3_Supporting

Functional studies support a damaging effect on protein function (Matreyek 2018 PMID: 29785012).

PP3_Supporting

Multiple lines of computational evidence predict a deleterious effect on the gene or gene product.

Computational missense predictions were performed using REVEL (Ioannidis 2016 PMID: 27666373).

PM5_Supporting

An alternative amino acid substitution at this codon has been reported as a likely pathogenic variant in one patient with Autism spectrum disorder with macrocephaly (Wu 2020 PMID: 31674007).
